# Professional Commitment, Satisfaction and Quality of Life of Nurses During the COVID-19 Pandemic in Konya, Turkey

**DOI:** 10.4314/ejhs.v32i2.20

**Published:** 2022-03

**Authors:** Şerife Didem Kaya, Nimetcan Mehmet, Kerem Şafak

**Affiliations:** 1 Health Management Department, Faculty of Health Science, Necmettin Erbakan University, Konya, Turkey; 2 Public Health Department, Faculty of Medicine, Ankara Yildirim Beyazit University, Ankara, Turkey; 3 Beyhekim Education ve Research Hospital, Konya, Turkey

**Keywords:** COVID-19, Professional Commitment, Professional Satisfaction, Professional Quality of Life, Nurse

## Abstract

**Background:**

The COVID-19 pandemic has negatively affected nurses. The aim of this study was to assess professional commitment, satisfaction and quality of professional life of nurses during the COVID-19 pandemic in Konya city, Turkey.

**Methods and Material:**

Cross-sectional study was conducted through online survey in March 2021 in Konya province of Turkey. Current working nurses from 30 public health facilities participated in the study. Standard questionnaire was used, the questionnaire has four components, the first part of questionnaire for characteristics of the participants, the second part was Nursing Professional Commitment Scale, the third part was regarding Professional Satisfaction Scale and the fourth part was related to Employees' Quality of Life Scale.

**Results:**

Total 407 nurses participated in the study, only 24% of the participants were male, 76% of them were married and 78% of them were undergraduate degree holders. About two-third (68%) of the nurses worked in COVID 19 treatment Unit serving/treatıng COVID 19 patients durıng this period. Some nurses (37%) have considered leaving their job during the Pandemic. Average scores of total nursing professional commitment 2.63±0.48. The nurses' working quality of life level was above average of total satisfaction. Comparing the age groups of the participants with the scale and sub-dimensions, a difference was found only with the Compassion fatigue sub-dimension (p<0.05). There is a positive relationship between total nursing professional commitment, compassion satisfaction and professional satisfaction.

**Conclusion:**

COVID-19 pandemic negatively affected nurses' professional commitment, professional satisfaction and quality of their professional life.

## Introduction

COVID-19 pandemic remains a public health emergency of international concern (PHEIC) (1). Starting from March 10, 2021, totally there have been 117.332.262 confirmed cases of COVID-19, including 2.605.356 deaths worldwide (2) while 2.821.943 confirmed cases and 29.227 deaths in Turkey (10 March 2021) (3). Since the beginning of the outbreak, health care providers have been showing more support, solidarity and gratitude than they ever have (4). Nurses are the front line healthcare workers fighting against COVID-19 daily and providing essential health services, they are striving to do their best to help their patients, in hospitals as well as in presently isolated areas (5). Many healthcare workers stopped showing up at work once COVID-19 hit the USA, due to the shortage of personal protective equipment (PPE) (6). Nurses have endured the brunt of the COVID-19 pandemic, more so than any other segment of the workforce (7).

The COVID-19 pandemic significantly exacerbated already existing burnout syndromes of nursing homes healthcare workers (8). The mental well-being of health professionals working on the front line is compromised in times of pandemic by presenting medium-high levels of anxiety, depression, nervousness and insomnia, and, to a lesser extent, stress (9). Being healthcare workers including a nurse in COVID-19 times can be a difficult profession, and is not free of risk. In addition to lack of protection, risk of contagion, and restructuring of services, the mental health of nurses in the performance of their duties has been harmed (10). The most important nurses` occupational values are worthwhile accomplishments, importance of professional challenge, diversity and interest in the job, personal growth and development and independence in their practice (11). It can be stated that improving quality of work life (QWL) requires a context-specific approach. For exploring the meaning of QWL and evaluating the specific significance of each QWL predictor for nurses in various countries, qualitative research is an invaluable tool (12).

As quality of work life has an important impact on attracting and retaining employees, it is necessary to pay more attention to the nurses' quality of work life and its affecting factors (13). COVID-19 had a negative effect on physical, mental and various aspects of quality of life of health care staff and led to increased fatigue and burnout, resulting in absence from work and its consequences (14). The aim of this study was to assess professional commitment, professional satisfaction and quality of professional life of nurses during the COVID-19 pandemic in Konya city, Turkey.

## Material and Methods

**Study design and sample**: A cross-sectional survey was conducted using a self-administered questionnaire among nurses in Konya city, Turkey. The data was collected through an online survey from March 1 to March 12, 2021. Nurses currently working in public health facilities during the COVID-19 pandemic participated in the study from 30 different public health facilities in the city. The nurses who were on parternity leave or admitted to hospital due to COVID-19 positive/infection were excluded during the study. The minimum sample size was 384 nurses according to sample size calculation (15) and 407 nurses responded to the questions.

**Study questionnaire**: The study questionnaire comprised of four parts, the first part of questionnaire for characteristics of the participants (14 questions), the second part was regarding Nursing Professional Commitment Scale (26 questions), the third part was regarding Professional Satisfaction Scale (scoring between 1–10) (16) and the fourth part was related Employees' Quality of Life Scale (30 questions). The Nursing Professional Commitment Scale is a 4 Point Likert Scale developed by other researchers (17) and its validity and reliability was done in Turkish (18). The answers to questions were assigned from 1 to 4 or strongly disagree to strongly agree on a four point Likert scale. (1=Strongly disagree, 2=Disagree, 3=Agree, 4=Strongly agree). The strongly disagree value was between 1–1.74; disagree value was 1.75–2.49; agree value was 2.50–3.24; strongly disagree value was 3.25–4.

The Employees' Quality of six scale is a five Point Likert Scale developed by other researchers (19) and its validity and reliability was tested in Turkish (20). The study used to measure respondent's response was Likert scale. The answers to questions were assigned from 0 to 5 or never to very often (0= Never, 1=Rarely, 2=Sometimes, 3=Frequently, 4=Usually, 5=Always) on a five point Likert scale. Value of never was between 0–0.82; for rare was 0.83–1.65; for sometimes was 1.66–2.49; for frequently was 2.50–3.33; usually was 3.33–4.16 and for always was 4.17–5.

The Turkish version of both scales were used in the study. The Cronbach's alpha was 0.88 for Desire to effort, 0.85 for Maintain to professional membership, 0.70 for Devotion to the goals and values, 0.92 for Total Nursing professional commitment, 0.90 for Compassion satisfaction, 0.72 for Burnout and 0.87 for compassion fatigue in this study.

**Data analysis**: Data analysis was done using Statistical Package for Social Sciences (SPSS) Version 22. We described socio-demographic distribution using frequency and percentage and for analysis of the work engagement, Job satisfaction and quality of work life of nurses during COVID-19 pandemic, we used the descriptive statistics, ANOVA, t-test, pearson correlation and multiple regression.

**Ethics consideration**: The study was approved by COVID-19 Scientific Research Studies Commission of Konya Provincial Health Directorate, Turkey Ministry of Health, and Meram Medical Faculty, Drug and Non-Medical Device Research Ethics Committee of Necmettin Erbakan University (Ethic Meeting No:117-Date: 02.09.2020). Prior to data collection, all study participants were given information on the study and assured that all data is confidential and will only be analyzed as aggregates. All respondents agreed to the consent form before participation in the survey.

## Results

The targeted sample and response rate we reached was hundred percent. Among the participants, 76% of them are female and married while 78% of them are under graduate degree holders (Table 1). The average working time of the participants in the nursing profession is 15 years. The highest participation rate is 40% of the employees in the operating room and other units (Table 2).

**Table 1:** Socio-demographic Characteristics of Participants (n=407)

Variable	Number	%
Gender		
Female	310	76
Male	97	24
Age Group (years)		
20–30	122	30
31–40	130	32
41–54 and above	155	38
Marial status		
Single	98	24
Maried	309	76
Education status		
High school	26	6
Undergraduate	318	78
Postgraduate	63	16

**Table 2:** Nurse's working unit and their thoughts

Variable	*Number*	*%*
Working unit		
Clinic	35	9
Emergency	14	3
Operating room	162	40
Polyclinic	13	3
Administrative units	21	5
Other	162	40

Protective equipment shortage		
Yes	168	41
No	239	59

Willingly choosing the profession		
Yes	309	76
No	98	24

Receiving additional payment from the ceiling		
Yes	304	75
No	103	25

Thoughts of resigning		
Yes	152	37
No	255	63

Negative affect of profession perspective in COVID-19 period		
Yes	152	37
No	255	63

Has a flexible working system been implemented during the COVID-19 Pandemic process?		
Yes	268	76
No	139	34

Working in COVID-19 unit		
Yes	278	68
No	139	32

	***number***	***Median***

COVID-19 working hours (weekly)	407	49

Working year in the profession	407	15

“Has a flexible working system been implemented during the COVID-19 Pandemic process?” 66% of the participants answered “yes” to the question. “Did you voluntarily choose the nursing profession?” 76% of the participants answered “yes”, 75% of the participants stated that they benefited from the regulation regarding the payment of the 3-month donor made during the COVID-19 Pandemic process from the ceiling. During the COVID-19 Pandemic period, 59% of the participants stated that they did not have any personal protective equipment shortage. Two thirds (68%) of the nurses reported that they worked in the unit serving COVID-19 Pandemic patients during this period. 63% of the participants answered “No” to the questions “Did the COVID-19 Pandemic process negatively affect your perspective on your profession?” and “Have you ever thought about resigning during the COVID-19 Pandemic process?” (Table 2).

Average scores of total nursing professional commitment 2.63±0.48; desire to effort 2.48±0.52; maintain to professional membership 2.54±0.63; devotion to the goals and values 2.86±0.52; compassion satisfaction 3.08±0.94; burnout 2.03±0.74; compassion fatigue 1.67±0.90; professional satisfaction scale 4.74±2.79. One Way ANOVA test was used to compare the age groups of the participants with the scale and its sub-dimensions. The findings are shown in Table 3.

**Table 3:** Comparison of the scales and sub-dimensions according to the age groups of the participants

	*Age (years)*	*Mean*	*SS*	*F*	*p*
Desire to effort	20–30	2.51	0.50	2.57	0.08
	31–40	2.44	0.53		
	41–54 and older	2.50	0.53		
	Total	2.48	0.52		
Maintain to professional membership	20–30	2.53	0.66	2.88	0.06
	31–40	2.51	0.65		
	41–54 and older	2.57	0.59		
	Total	2.54	0.63		
Devotion to the goals and values	20–30	2.97	0.53	0.41	0.67
	31–40	2.77	0.54		
	41–54 and older	2.86	0.48		
	Total	2.86	0.52		
Nursing professional commitment	20–30	2.67	0.50	1.62	0.20
	31–40	2.57	0.51		
	41–54 and older	2.64	0.45		
	Total	2.63	0.48		
Compassion satisfaction	20–30	3.12	0.88	0.17	0.84
	31–40	2.97	0.95		
	41–54 and older	3.14	0.98		
	Total	3.08	0.94		
Burnout	20–30	2.04	0.75	0.65	0.53
	31–40	2.11	0.73		
	41–54 and older	1.95	0.75		
	Total	2.03	0.74		
Compassion fatigue	20–30	1.71	0.81	6.26	0.00
	31–40	1.59	0.83		
	41–54 and older	1.72	1.03		
	Total	1.67	0.90		
Professional satisfaction	20–30	4.26	2.54	2.86	0.06
	31–40	4.63	2.82		
	41–54 and older	5.21	2.90		
	Total	4.74	2.79		

Since there is no homogeneous distribution among the groups in the variables of gender, marital status, education status, working unit of the participants, these variables were excluded from the analysis.

Comparing the age groups of the participants with the scale and sub-dimensions, a difference was found only with the Compassion fatigue subdimension (p<0.05).

The average burnout values (2.11±0.73) of the nurses in the 31–40 age group are higher than the other age groups. The average value of compassion fatigue (1.59±0.83) in the 31–40 age group was lower than the other age groups. It is seen that the average of professional satisfaction increases with age (Table 3).

Participants “Have you worked in the unit serving COVID-19 (Coronavirus) patients?” and “Did the COVID-19 process negatively affect your perspective on your profession?” that test was used to compare the answers they gave to the question with the scale and its sub-dimensions. The findings are shown in Table 4.

**Table 4:** Nurses who worked in COVID-19 servises VS. who did not work during the COVID-19 pandamic

*Have you worked in the unit serving COVID-19 patients?* *n= Yes: 278 No: 129*		*Mean*	*SS*	*t*	*p*
Desire to effort	Yes	2.44	0.52		0.30
	No	2.56	0.53	-2.16	
Maintain to professional membership	Yes	2.49	0.65		0.20
	No	2.65	0.58	-2.36	
Devotion to the goals and values	Yes	2.85	0.54		0.36
	No	2.90	0.46	-0.99	
Nursing professional commitment	Yes	2.59	0.49		0.03
	No	2.71	0.46	-2.16	
Compassion satisfaction	Yes	3.04	0.95		0.26
	No	3.16	0.94	-1.13	
Burnout	Yes	2.06	0.75		0.14
	No	1.95	0.72	1.50	
Compassion fatigue	Yes	1.68	0.92		0.92
	No	1.67	0.86	0.10	
Professional satisfaction	Yes	4.64	2.77	-1.08	0.28
	No	4.96	2.83		
***Did the COVID-19 process negatively affect your perspective on*** ***your profession? n= Yes: 240 No: 167***		***Mean***	***SS***	***t***	***p***
Desire to effort	Yes	2.30	0.50	-9.05	0.00
	No	2.74	0.45		
Maintain to professional membership	Yes	2.31	0.57	-10.10	0.00
	No	2.88	0.56		
Devotion to the goals and values	Yes	2.79	0.53	-3.63	0.00
	No	2.97	0.48		
Nursing professional commitment	Yes	2.47	0.45	-8.94	0.00
	No	2.86	0.44		
Compassion satisfaction	Yes	2.85	0.90	-6.13	0.00
	No	3.41	0.91		
Burnout	Yes	2.33	0.69	11.05	0.00
	No	1.60	0.60		
Compassion fatigue	Yes	1.97	0.95	9.30	0.00
	No	1.25	0.62		
Professional satisfaction	Yes	3.65	2.39	-10.71	0.00
	No	6.31	2.58		

“Have you worked in the unit that serves COVID-19 patients?” When the answer to the question was compared with the scale and its sub-dimensions, a significant difference was found only with total nursing professional commitment (p<0.05). The average values of total professional commitment (2.71±0.46) of nurses who do not work in the unit serving COVID-19 patients were higher than Than other employees who do not serve COVID19 employee. There was no difference with the other groups (p>0.05) (Table 4).

“Did the COVID-19 process negatively affect your perspective on your profession?” when the answers given to the question and the scale and its sub-dimensions were compared, a significant difference was found between the whole scale and its sub-dimensions (p<0.05) (Table 4).

The average values of nursing professional commitment, desire to effort, maintain professional membership, devotion to the goals and values, compassion satisfaction and professional satisfaction were higher than the other group. But burnout and compassion fatigue was lower.

The relationship between nursing professional commitment, professional quality of life and professional satisfaction scale dimensions are given in Table 5. According to the table; professional quality of life scale and compassion satisfaction” dimension has positive relationship and very high total with nursing professional commitment scale score and “desire to effort” dimension (respectively r=0.75, r=0.70; p<0.01). Professional quality of life scale “compassion satisfaction” dimension has positive relationship and median with “maintain to professional membership”, “devotion to the goals and values” and “professional satisfaction scale” score (respectively r=0.62, r=0.64, r=0.51; p<0.01). Professional quality of life scale “burnout” dimension has negative relationship and median with total nursing professional commitment scale score and all its dimensions (respectively r=-0.61, r=-0.56, r=-0.60, r=-0.41, r=-0.63; p<0.01). Professional quality of life scale “compassion fatigue” dimension has negative relationship and median with total nursing professional commitment scale, maintain to professional membership and professional satisfaction scale score (respectively r=-0.30, r=-0.39, r=-0.37; p<0.01). But it has negative relationship and weak with desire to effort and devotion to the goals and values score (respectively r=-0.24, r=-0.12; p<0.01). Professional satisfaction scale has positive relationship and median with nursing professional commitment scale score and all its dimension (respectively r=0.62, r=0.62, r=0.66, r=0.32; p<0.01).

**Table 5:** Relationship between nursing professional commitment professional quality of life and all its dimensions and professional satisfaction

		*Compassion* *satisfaction*	*Burnout*	*Compassion* *fatigue*	*Professional* *satisfaction* *scale*
Nursing professional commitment	r*	0.75**	-0.61**	-0.30**	0.62**
Desire to effort	r*	0.70**	-0.56**	-0.24**	0.62**
Maintain to professional membership	r*	0.62**	-0.60**	-0.39**	0.66**
Devotion to the goals and values	r*	0.64**	-0.41**	-0.12**	0.32**
Professional satisfaction	r*	0.51**	-0.63**	-0.37**	1

** Correlation is significant at the 0.01 level (2-tailed).

* weak ≤ 0.29. 0.30 ≤ median ≥ 0.69. high ≥ 0.70 (21)

Multiple regression analysis was performed to predict nurses' professional commitment based on their perceptions of professional satisfaction and professional quality of life sub-dimensions (compassion satisfaction, burnout, compassion fatigue).

As seen in Table 6, the change in the total nursing professional commitment variable is explained by 65% compassion satisfaction, burnout, compassion fatigue and professional satisfaction. The F value is 182.30, which indicates that our model is meaningful as a whole (p=0.000). There is a positive relationship between total nursing professional commitment. Compassion satisfaction and professional satisfaction included in the model. From the t value, it is seen that this relationship is statistically significant (respectively t=13.07, t=13.56, t=7.74; p=0.000).

**Table 6:** Determining the effects of compassion satisfaction, burnout, compassion fatigue and professional satisfaction on total nursesing professional commitment

	*B*	*Standard error*	*Beta*	*t*	*p*	*VIF*
Constant	1.56	0.12		13.07	0.000	
Compassion satisfaction	0.29	0.02	0.57	13.56	0.000	2.02
Burnout	-0.01	0.04	-0.02	-0.36	0.72	3.92
Compassion fatigue	-0.03	0.02	-0.05	-1.22	0.22	2.14
Professional satisfaction	0.05	0.01	0.30	7.74	0.000	1.70
R= 0.803	R^2^= 0.65	F= 182.30	p= 0.000		Durbin Watson=2.03	

Dependent variable: Total nursing professional commitment

## Discussion

Most of the participants stated that they chose the nursing profession voluntarily (76%), benefited from the top level additional payment made during the pandemic period (75%), did not have protective equipment problems (59%) benefited from the flexible working system but (66%), of the nurses' view of their profession was negatively affected during the COVID-19 process” (37%), was the same with the rate of the statement that “they thought to be resigning” (37%). It can be seen that nurses, who had a negative view of their profession during the COVID-19 pandemic period, also had the idea of resigning. However, during this period, with the circular dated 27.10.2020 and numbered 60438742-929 published by the Ministry of Health, resignation, retirement and annual leave bans were imposed on health workers.

The rate of those who willingly chose the nursing profession (76%) and the rate of those who do not intend to resign (63%) are over 50%. This situation shows that those who willingly choose this profession can continue it even under difficult conditions. Practices such as flexible working and top level additional payment are thought to positively affect the willingness to continue the nursing profession. It can also be said that the use of protective equipment has an effect on reducing anxiety and disquiet levels.

Considering that the weekly working time is normally 40 hours, that the average working hour of the nurses is 49 hours indicates that the nurses work overtime. During the pandemic period, with the circulars issues in Turkey, flexible working hours of public personnel have been introduced. However, employees of the Ministry of Health and some ministries' service were needed to be continuous and have been excluded from the scope of flexible working hours. Health workers, who were in an intense struggle with the epidemic, which continued without reducing its impact and reached its peak in some periods, both had to work harder than other public employees and faced a greater risk. This situation negatively affects the professional satisfaction, nursing professional commitment and quality of life of the nurses. In Danacı's (22) study, for the relationship between weekly working hours and professional satisfaction; the professional satisfaction of nurses working more than 40 hours per week was found to be lower than the professional satisfaction of nurses working 40 hours or less per week in freedom, activity, progress and working conditions factors.

It was found that total nursing professional commitment average (2.63±0.48) of the nurses was “I agree”, the sub-dimension of willingness to make effort (2.48±0.52) was “I don't agree”; the sub-dimension of maintaining professional membership (2.54±0.63) was “I agree” and the sub-dimension of devotion to the goals and values was (2.86±0.52) “I agree”. All values were above average. Nurses believe in and accept the values of their profession, they are willing to continue their professional membership and improve themselves in the professional field. However, the findings can be interpreted as that they are not very willing to make an effort while doing all of these. Looking at the studies carried out before the pandemic, “total nursing professional commitment” average values of the nurses was found above intermediate level as 2.82 in Çetınkaya et al.,(18); 3.19±0.62 in Kaya and Zerenler (23) and 3.60±0.64 in Cihangiroğlu et al., (24). It can be said that the value of nursing professional commitment decreased partially with the pandemic.

When the average values of the subdimensions of the nurses' working quality of life scale are examined, it was found that the average value of the professional satisfaction subdimension (3.08±0.94) was “frequently”; average value of burnout sub-dimension (2.03±0.74) was “sometimes”, the average value of the compassion fatigue sub-dimension (1.67±0.90) was “sometimes”. It is understood from these results that nurses are often satisfied as helpers. However, they stated that they sometimes had difficulties in coping with the problems that occur in their working life, they fell into despair and were exhausted. When faced with stressful events in their working lives, we can say that nurses sometimes experience negative symptoms. It is important for the protection of health to know these symptoms of burnout and compassion fatigue in nurses and to diagnose them early, to know the protective factors that prevent their occurrence and the risk factors that facilitate them (25). Otherwise, nurses will need professional help. We can say that the effect of the pandemic period also increases these values. The values in the study conducted by Polat and Erdem (26) on physicians and nurses are professional satisfaction (3.880±0.644), burnout (2.280±0.611), compassion fatigue (2.552±0.625). Kaya (27) stated that 25.6% of nurses-midwives have poor general quality of life.

Therefore, it is of vital importance to increase professional satisfaction in health institutions in terms of preventing compassion fatigue and burnout. In the study, the professional satisfaction average of the nurses was also below the intermediate level (4.74±2.79). In the study conducted by Senek et al. (28) in England, it was observed that the professional satisfaction level of most of the nurses was low. It can be concluded that the difficult working process during the pandemic period negatively affects the nursing professional commitment, professional life quality and professional satisfaction of nurses. In addition, the anxiety level perception of nurses with a high perception of organizational and social support and resilient nurses about COVID-19 was found to be lower (29).

In the study, when the age groups of the nurses were compared with the sub-dimensions of professional satisfaction, nursing professional commitment and quality of life, a difference was found only in the compassion fatigue sub-dimension of the quality of life scale (p< 0.05). The average compassion fatigue value (1.59±0.83) of the nurses in the 31–40 age group was lower than the compassion fatigue average value of the nurses in the 20–30 age group (1.71± 0.81) and the 41–54 age group (1.72±1.03). In the study of Berger et al. (30), it was also found that younger nurses had less compassion fatigue. On the other hand, in the study of Denk (31), no difference was found between the age variable and the subdimensions of life quality. In the study, no difference was found between age groups and burnout sub-dimension. However, it is seen that the average burnout values (2.11±0.73) of the nurses in the 31–40 age group are higher than the other age groups. It can be concluded that this age group experiences burnout more than other age groups, but they can tolerate the symptoms of compassion fatigue more. When other studies on burnout were examined, a difference was found between the age variable and burnout in the study of Bilben (32). Burnout decreases with increasing age. In the study of Sacco et al. (33), they found that nurses in the 40–49 age group experienced burnout more. In the study, it was seen that the average of professional satisfaction increased with age. It is thought that this may be due to the fact that nurses in younger age groups are generally employed in more risky places.

In the study, a difference was found only between nurses working in units serving COVID-19 patients and the total nursing professional commitment scale (p<0.05). The average values of nurses who do not work in units serving COVID-19 patients in terms of nursing professional commitment, willingness to make effort, maintaining professional membership, belief in goals and values, and professional satisfaction were found to be higher than the other group. Their compassion fatigue and burnout average values were lower. In the study conducted by Wu et al. (34) the burnout levels of physicians and nurses working in pandemic services (13%) were lower than those working in other services (39%). It can be assumed that nurses' knowing and accepting potential risky situations in their professional lives causes them to be physically and psychologically stronger.

When the answer given to the question about “whether the COVID-19 process affected the professional perspective negatively” was compared with the nursing professional commitment and its sub-dimensions, the subdimensions of life quality in working life and professional satisfaction, a significant difference was found between the whole scale and its subdimensions (p<0.05). The average values of nurses whose perception of their profession is not negative during the COVID-19 pandemic in terms of nursing professional commitment, willingness to make effort, maintain professional membership, belief in the goals and values, compassion satisfaction and professional satisfaction are higher than the other group. In addition, these nurses experience less burnout and lower levels of compassion fatigue. The study was conducted by Liu et al. (35), reported that the pandemic period increased burnout in healthcare workers in moderate and severe levels. The negative copingstyles of physicians and nurses working in a high infection zone can negatively affect burnout. Ways to reduce burnout, such as reducing nurses' workloads and providing better protection from the virus, should be used.

There is a positive and high relationship between total nursing professional commitment, professional satisfaction and compassion satisfaction (r=0.75, r=0.62; p<0.01 respectively); and there is a negative and intermediate level relationship between burnout and compassion fatigue (r=-0.61, r=-0.30; p<0.01 respectively). A positive and high relationship was found between professional satisfaction and compassion satisfaction (r=0.51; p<0.01), and a negative and intermediate level relationship was found between burnout and compassion fatigue (r=-0.63, r=-0.37 respectively; p<0.01). Total nursing professional commitment is explained by 65% compassion satisfaction, burnout, compassion fatigue and professional satisfaction. Durmus et al. (36) found a strong negative significant relationship between nurses' burnout and quality of life (p<0.001).

It was determined that nurses experienced burnout and their quality of life was low, and some variables negatively affected their quality of life. The pandemic process and difficult working conditions can be shown as examples of these negative effects. Karakose and Bozbeyikli (37) stated that total organizational commitment explained 22.2% of the variance regarding the scores obtained from the burnout sub-dimension of quality of life, 13.2% of the variance regarding the scores obtained from the compassion fatigue sub-dimension and 26.5% of the variance regarding the scores obtained from the professional satisfaction sub-dimension. This study was conducted in several hospitals of Konya city so the findings of the study may not show the whole nurses situation in the country.

In conclusion, Around one thirds (37%) of nurses have considered leaving their job during the Pandemic. Nurses' professional commitment was more than average in this study, the total mean score was 2.63±0.48 (the average score was 2.48±0.52 for willingness to make effort subscale; subscale of continuing professional membership was 2.54±0.63; belief in goals and values was 2.86±0.52). The nurses' working quality of life level was above average of total satisfaction.

The nurses exhibited a medium level of job satisfaction (4.74±2.79). There was a positive and high correlation between total commitment to the nursing profession and professional satisfaction (r=0.75, r=0.62; p<0.01, respectively); There is a negative relationship (r=-0.61, r=-0.30, p<0.01, respectively) between burnout and compassion fatigue. There was a positive and high (r=0.51; p<0.01) relationship between professional satisfaction and compassion satisfaction; and there was a negative and median level (r=-0.63, r=-0.37; p<0.01, respectively) relationship between burnout and compassion fatigue. Around two thirds (65%) of the total commitment to the nursing profession was explained by the quality of life burnout, spouse fatigue, professional satisfaction and compassion satisfaction.
